# Enhanced Wnt Signalling in Hepatocytes is Associated with *Schistosoma japonicum* Infection and Contributes to Liver Fibrosis

**DOI:** 10.1038/s41598-017-00377-4

**Published:** 2017-03-22

**Authors:** Qi Wang, Xin Chou, Fei Guan, Zhengming Fang, Shengjun Lu, Jiahui Lei, Yonglong Li, Wenqi Liu

**Affiliations:** Department of Parasitology, School of Basic Medicine, Tongji Medical College, Huazhong University of Science and Technology, Wuhan, Hubei P.R. China

## Abstract

Liver fibrosis is the most serious pathology caused by *Schistosoma japonicum* infection, which arises when schistosome eggs are deposited in the liver. Eosinophils, macrophages and hepatic stellate cells (HSCs) have been identified as major cellular contributors to the development of granulomas and fibrosis, but little is known about the effects of hepatocytes on granuloma formation. Here, we found that the levels of Wnt signalling-related molecules, transforming growth factor β (TGF-β) and connective tissue growth factor (CTGF) in hepatocytes were markedly elevated after *S. japonicum* infection. Liver fibrosis was exacerbated when exogenous Wnt3a was introduced, but was alleviated when Wnt signalling was suppressed by DKK1, accompanied by the reduced expression of TGF-β and CTGF in hepatocytes. These results indicate that the hepatocytic expression of TGF-β and CTGF is mediated by Wnt signalling. Additionally, the hepatocytes isolated from infected mice promoted the activation of primary HSCs *in vitro*, however, this effect was not observed when hepatocytes from DKK1 treated *S. japonicum*-infected mice was employed in the co-culture system. Our findings identify a novel pro-fibrogenic role of hepatocytes in schistosomiasis-induced liver fibrosis that is dependent on Wnt signalling, which may serve as a potential target for ameliorating hepatic fibrosis caused by helminths.

## Introduction

Schistosomiasis is a helminth infectious disease that is estimated to threaten approximately 200 million people in more than 74 countries where it is endemic^1, 2^. Hepatic fibrosis is the most serious pathological effect of *S. japonicum* infection, and it arises when schistosome eggs are deposited in the host’s liver^1, 3, 4^. The immune reaction and inflammatory response caused by soluble egg antigen (SEA) released from schistosome eggs can alter the intrahepatic microenvironment. This process consequently stimulates the trans-differentiation of hepatic stellate cells (HSCs) into activated myofibroblasts and the excessive deposition of collagen and other extracellular matrix (ECM) components in the liver, which results in the formation of granulomas and hepatic fibrosis surrounding the schistosome eggs^5, 6^. HSCs are believed to be a principal trigger of collagen deposition in the liver, and they play a major role in ECM remodelling in schistosomiasis^4, 7^. The activation of HSCs is central to hepatic fibrosis progression^8, 9^. An egg-evoked Th2 response whereby interleukin 4 (IL-4) and IL-13 dominate has been recognized as being pivotal to the initiation of schistosome-induced fibrogenesis via the activation of HSCs and stimulation of collagens. TGF-β produced by activated HSCs or neighbouring liver cells has also been identified as a central pro-fibrogenic mediator^4^, which is associated in part with its downstream effector connective tissue growth factor (CTGF). These well-known pro-fibrotic factors may sequentially elicit diverse intracellular pathways such as the mitogen-activated protein kinase (MAPK), TLR4-MyD88-NF-kB, Hippo or Wnt pathways, which contribute to the regulation of HSC trans-differentiation^10–13^.

Wnt signalling is an evolutionarily conserved, highly complex pathway that is critical for development, differentiation and cellular homeostasis. The protein β-catenin is the central player of the canonical Wnt pathway, and it plays important roles in the development, regeneration and metabolism of the liver as well as in the maintenance of normal function of the adult liver^13–16^. More recently, Wnt signalling has been reported to be involved in liver fibrosis^15^. Several Wnt ligands (Wnt3a, Wnt4, Wnt5a and Wnt10b) and the Wnt receptors [frizzled1 (Fz1), frizzled2 (Fz2)] were found to be upregulated in activated hepatic stellate cells compared with quiescent hepatic stellate cells^17^. Moreover, higher levels of nuclear β-catenin and T-cell factor (TCF) DNA binding proteins were observed in activated HSCs^17^. The suppression of Wnt signalling may inhibit the activation of HSCs and liver fibrosis^17^, and Wnt signalling can enhance the activation and survival of human hepatic stellate cells^18^. Together, these findings suggest that canonical Wnt signalling may be an emerging key pathway in hepatic stellate cell activation and liver fibrosis.

As a critical organ involved in metabolic function, the liver consists of approximately 80% parenchymal hepatocytes and 20% non-parenchymal cells, such as stellate cells, sinusoidal endothelial cells, Kupffer cells, biliary epithelial cells and recruited peripheral immune cells^19^. During the development of schistosomiasis-induced liver fibrosis, ongoing immune reactions and inflammatory responses are both thought to contribute to the pathological changes in the liver microenvironment, and such changes may affect the fate of all cells in the liver. Excessive activation of stellate cells is the substantial and valuable core event in the pathological damage. As a major functional portion, liver parenchymal cells are inevitably involved in liver injury and fibrotic regeneration. It is still unknown whether *S. japonicum* eggs affect the fate of hepatocytes, and the role of hepatocytes in liver fibrosis caused by *S. japonicum* infection remains to be determined. It has been reported that six Wnt proteins (Wnt2, Wnt4, Wnt5a, Wnt5b, Wnt9a and Wnt9b) and five Fz molecules (Fz2, Fz4, Fz6, Fz7 and Fz8) are expressed in hepatocytes isolated from normal liver tissue^20^. This finding suggests that hepatocyte-derived Wnt molecules have the potential to activate Wnt signalling in hepatocytes or other resident liver cells.

In this study, BALB/c mice were infected with *S. japonicum* to investigate the expression of Wnt-related molecules and the fibrotic factors TGF-β and CTGF in hepatocytes. We also evaluated the effects of Wnt signalling in hepatocytes on HSC activation and schistosomiasis-induced liver fibrosis. The relationship between Wnt signalling and fibrotic factors (TGF-β and CTGF) in hepatocytes was also examined by inhibiting Wnt-induced Fz-LRP5/6 complex formation^17^. Our results reveal a previously unknown pro-fibrogenic function of hepatocytes in which Wnt signalling in hepatocytes appears to promote liver fibrogenesis resulting from *S. japonicum* infection by inducing the expression of TGF-β and CTGF.

## Results

### *S. japonicum* infection accelerates the expression of Wnt signalling molecules in hepatocytes

**Figure 1:**
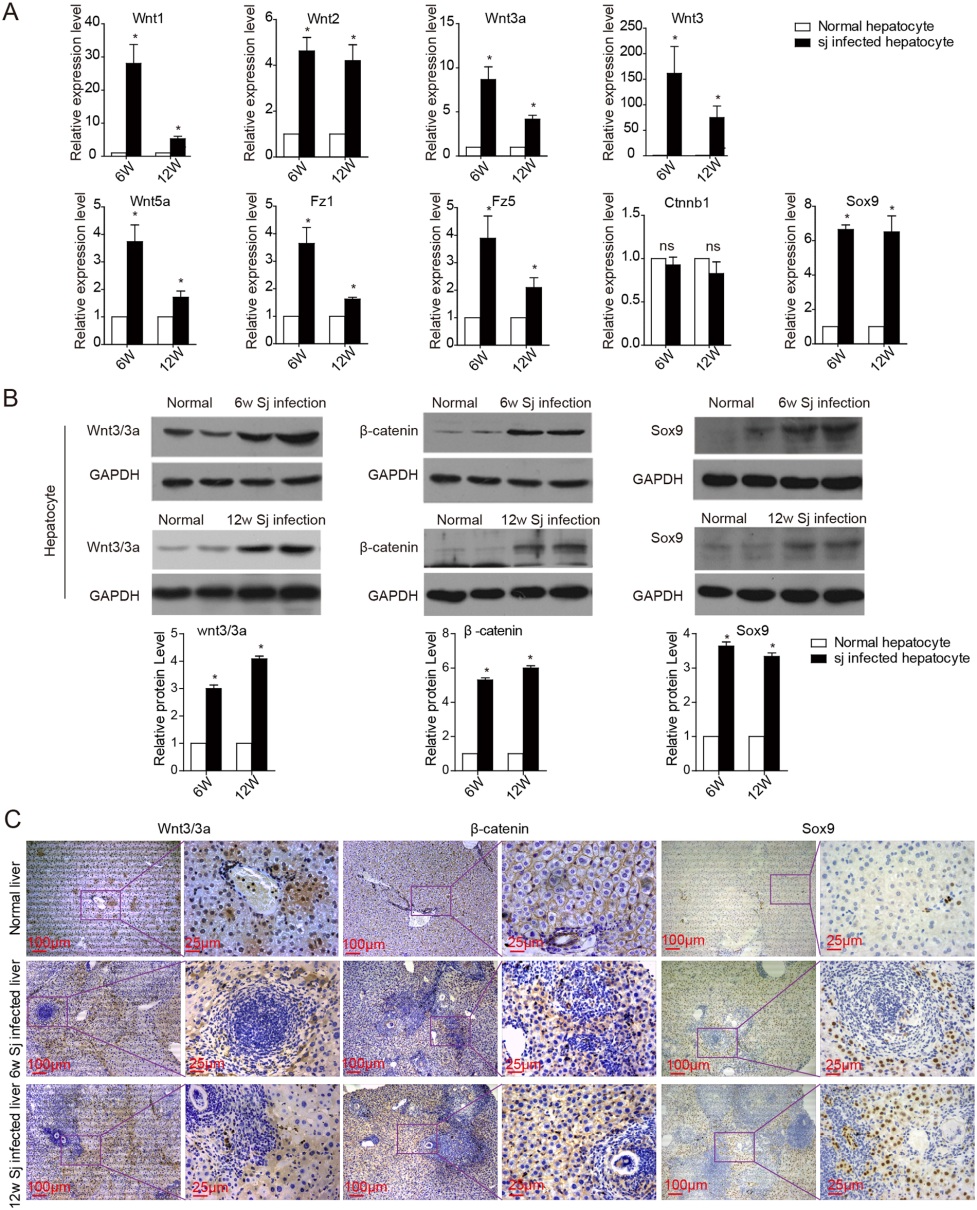
*S. japonicum* infection accelerates the expression of Wnt signalling-related molecules in hepatocytes. Primary hepatocytes were isolated from normal mice and mice after 6 and 12 weeks of *S. japonicum* infection. (**A**) Real-time PCR analysis of Wnt1, Wnt2, Wnt3, Wnt3a (canonical molecules), and Wnt5a (noncanonical); Fz1 and Fz5 (receptors of Wnt); and the Wnt signalling target genes β-catenin and Sox9. (**B**) The expression of Wnt3/3a, β-catenin, and Sox9 was detected by western blotting 6 or 12 weeks after infection. (**C**) Liver sections were immunohistochemically stained for Wnt3/3a, β-catenin, and Sox9. Sj, *S. japonicum*. Scale bar, 100 μm (left) and 25 μm (right). Data represent the mean ± SEM; n = 5 (*P < 0.05). ns, no significance.

**Figure 2:**
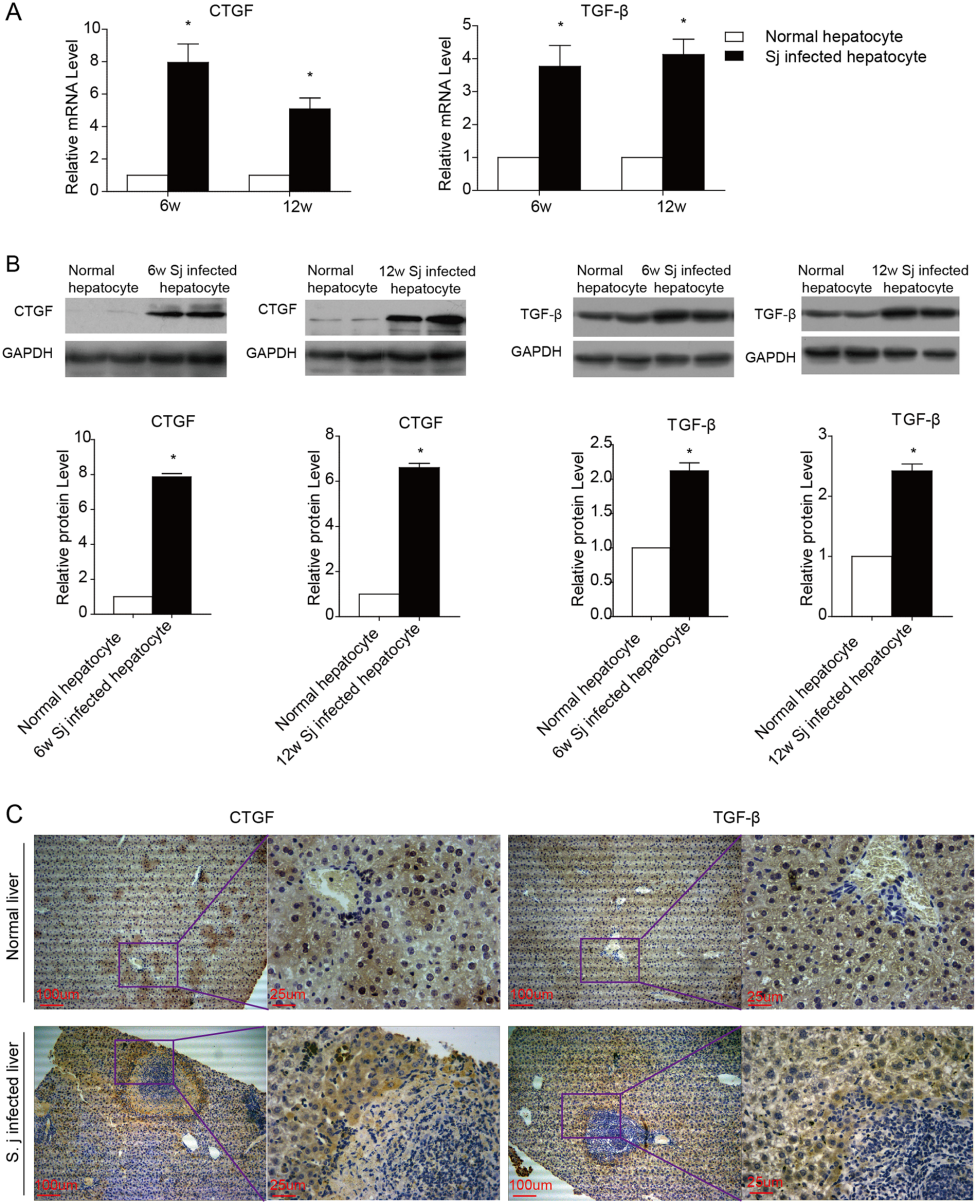
*S. japonicum* infection promotes the expression of fibrotic factors in hepatocytes. (**A** and **B**) Real-time PCR and western blotting were used to examine the expression of CTGF and TGF-β in primary hepatocytes purified from schistosomiasis-infected livers 6 and 12 weeks after infection. (**C**) Liver sections were immunohistochemically stained for CTGF and TGF-β. Sj, *S. japonicum*. Scale bar, 100 μm (left) and 25 μm (right). Data represent the mean ± SEM; n = 5 (*P < 0.05).

BALB/c mice were infected with 25 cercariae of *S. japonicum* through percutaneous exposure of the abdominal skin, and the hepatocytes were successfully isolated from the liver (Supplementary Fig. 1A,B) at the indicated time. The infected mice exhibited increased mRNA levels of canonical (Wnt1, Wnt2, Wnt3, and Wnt3a) and noncanonical (Wnt5a) Wnt molecules, receptors (Fz1 and Fz5) and the Wnt signalling target gene Sox9 in hepatocytes at 6 and 12 weeks post-infection (Fig. 1A). No significant differences in β-catenin were observed at the mRNA level (Fig. 1A). In addition, there was an increase in the protein levels of Wnt3/3a, β-catenin and Sox9 in the hepatocytes after *S. japonicum* infection (Fig. 2B,C and Supplementary Fig. 9). These results suggest that both canonical and non-canonical Wnt signalling in hepatocytes was obviously induced after egg deposition, and this signalling was involved in the development of schistosomiasis.

### *S. japonicum* infection drives the expression of the fibrotic factors CTGF and TGF-β in hepatocytes

CTGF is critically involved in fibrotic diseases by regulating fibroblast growth, and TGF-β is well known to stimulate HSCs in liver fibrosis. We examined the expression of CTGF and TGF-β in hepatocytes during schistosomiasis-induced liver fibrosis. After 6 and 12 weeks of *S. japonicum* infection, the mRNA levels of CTGF and TGF-β were found to be elevated in infected hepatocytes compared with normal hepatocytes (Fig. 2A), and the protein levels were consistent with the mRNA levels (Fig. 2B). Furthermore, CTGF and TGF-β were more often detected in hepatocytes in close proximity to areas containing egg granulomas (Fig. 2C and Supplementary Fig. 10). These findings indicate that hepatocytes may contribute to granuloma formation and the fibrosis process by driving the activation of HSCs or directly affecting schistosome-induced extracellular matrix accumulation.

### The successful transfection and expression of recombinant lentiviruses (Lv-EGFP, Lv-Wnt3a-EGFP and Lv-Dkk1-EGFP) in the livers of mice infected with *S. japonicum*

To investigate the effects of Wnt signalling on schistosomiasis-induced liver fibrosis, two lentiviral vectors loaded with Wnt3a (Lv-Wnt3a-EGFP) or DKK1 (Lv-DKK1-EGFP) were successfully established and injected via the tail vein into mice infected with *S. japonicum* for 6 weeks and into normal mice. Two weeks later, the liver tissues were harvested, and frozen sections were prepared to observe the expression of EGFP by fluorescence microscopy. As shown in Supplementary Fig. 2A and B, Lv-EGFP, Lv-Wnt3a-EGFP and Lv-DKK1-EGFP were effectively expressed in the livers of normal and *S. japonicum*-infected mice. These recombinant lentiviruses did not cause any obvious pathological changes in the livers when injected into normal mice (Supplementary Fig. 2A).

### Wnt3a-lentivirus transduction exacerbates schistosomiasis-induced liver fibrosis

**Figure 3:**
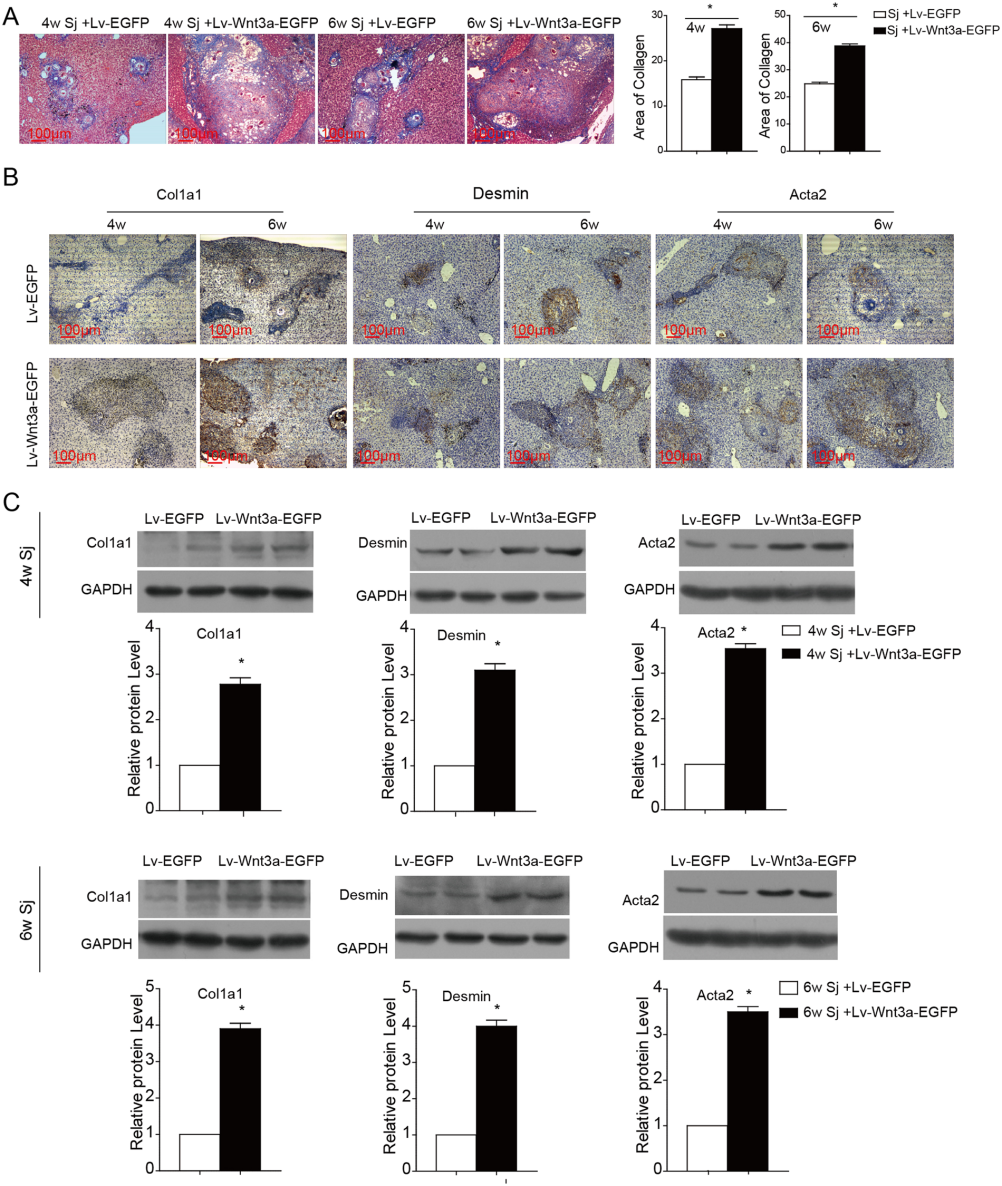
Wnt3a overexpression exacerbates liver fibrosis caused by *S. japonicum* infection. Mice received either Lv-EGFP or Lv-Wnt3a-EGFP after *S. japonicum* infection for 4 or 6 weeks. (**A**) Masson’s trichrome staining of the liver sections was used to measure collagen fibre deposition, and the ratio of the collagen-stained area to the total area was also quantified. (**B**,**C**) Hepatic expression of fibrotic markers (Col1a1, Desmin, and α-SMA/Acta2) was assessed by immunohistochemical staining and western blotting. Sj, *S. japonicum*. Scale bar, 100 μm. Data represent the mean ± SEM; n = 5 (*P < 0.05).

After 4 or 6 weeks of *S. japonicum* infection, either Lv-Wnt3a-EGFP or Lv-EGFP was injected into the tail vein. Two weeks later, the liver tissues were obtained for Masson’s trichrome staining to observe collagen fibre deposition. As shown in Fig. 3A, the percentage of area that was positive for collagen staining in the livers of the Lv-Wnt3a-EGFP-treated mice was larger than that of the Lv-EGFP-treated mice. In addition, immunohistochemical staining and western blot analysis were used to assess the expression of α-SMA/Acta2, desmin and Col1a1. As a result of the over expression of Wnt3a (Fig. 3B,C), the protein expression of α-SMA/Acta2, desmin and Col1a1 in the livers was increased (Supplementary Fig. 11). These results confirm the relevance of the activation of Wnt signalling to schistosome-induced fibrosis.

### DKK1-lentivirus transduction inhibits schistosomiasis-induced liver fibrosis

**Figure 4:**
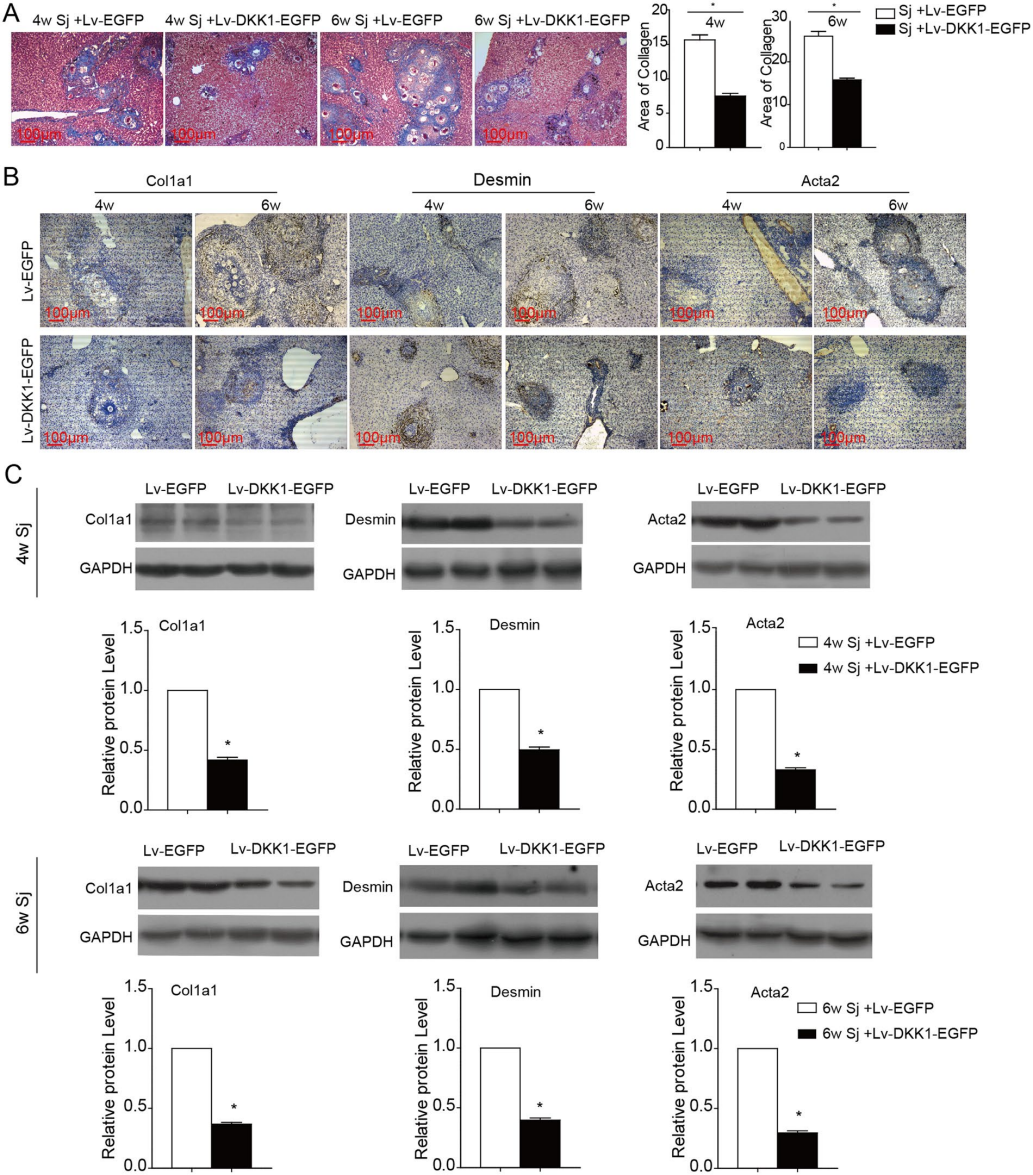
DKK1 transduction protects against schistosomiasis-induced liver fibrosis. Lv-DKK1-EGFP was injected via the tail vein to suppress Wnt signalling in the liver after *S. japonicum* infection for 4 or 6 weeks. (**A**) Masson’s trichrome staining was used to measure collagen fibre deposition, and the ratio of the collagen-stained area to the total area was also quantified. (**B**,**C**) Fibrotic markers (Col1a1, desmin, and α-SMA/Acta2) in the liver were assessed by immunohistochemical staining and western blotting. Sj, *S. japonicum*. Scale bar, 100 μm. Data represent the mean ± SEM; n = 5 (*P < 0.05).

DKK1 can specifically block the canonical Wnt pathway by binding to the low-density lipoprotein receptor-related protein (LRP) 5/6 component of the Wnt-induced Fz-LRP5/6 complex^17^. We investigated the effects of DKK1 on schistosomiasis-induced liver fibrosis as a result of the suppression of Wnt signalling through injection of Lv-DKK1-EGFP 4 or 6 weeks after *S. japonicum* infection. The results showed that the collagen deposition and liver fibrosis caused by *S. japonicum* infection were markedly alleviated after DKK1 treatment (Fig. 4A), which was accompanied by a decrease in the expression of α-SMA/Acta2, desmin and Col1a1 (Fig. 4B,C and Supplementary Fig. 12). These findings (Figs 3 and 4) indicate that Wnt signalling could aggravate schistosomiasis-induced liver fibrosis by facilitating the activation of HSCs and the synthesis of the ECM.

### DKK1 transduction inhibits the expression of CTGF and TGF-β

**Figure 5:**
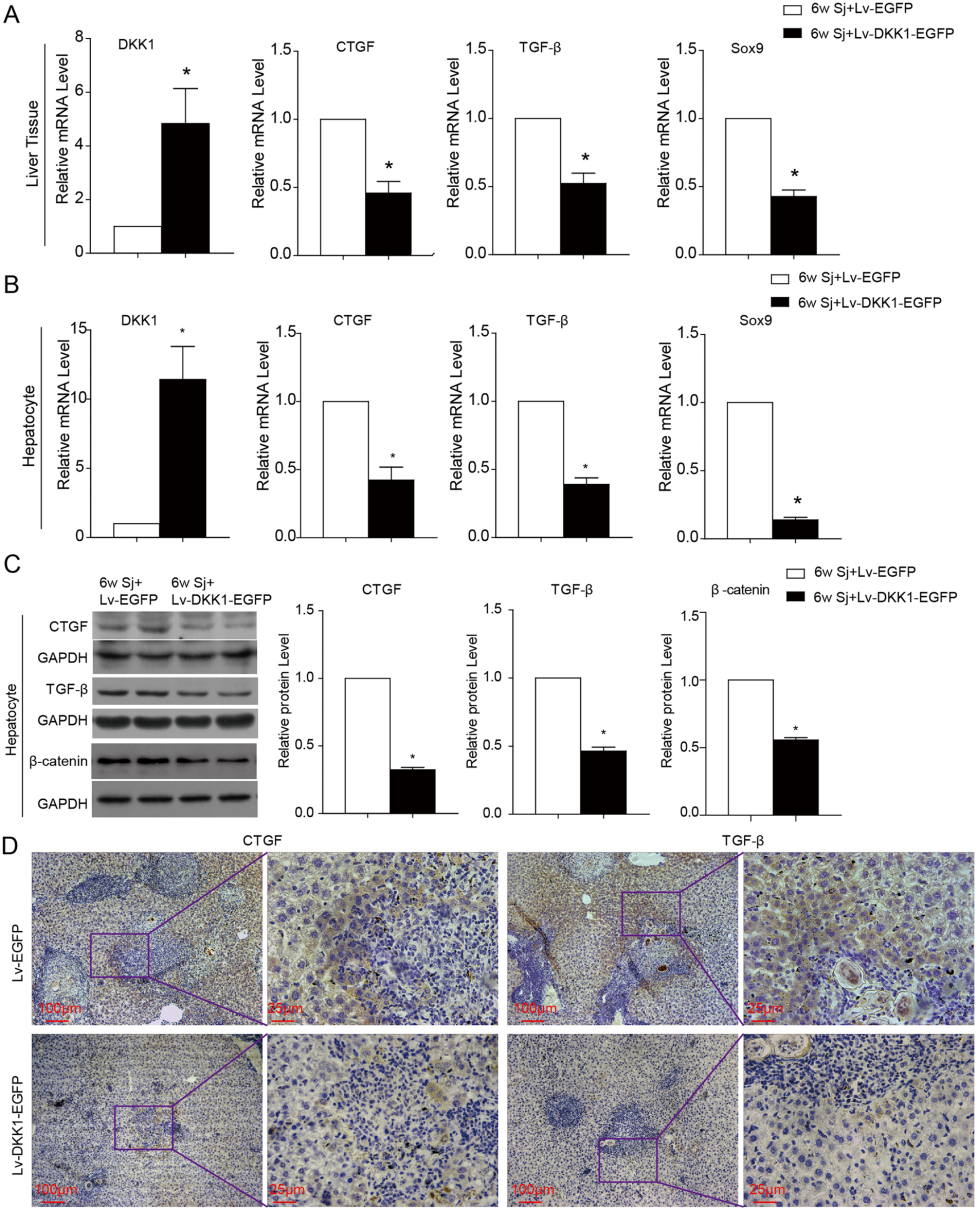
Dkk1 transduction inhibits the expression of CTGF and TGF-β in hepatocytes. The liver tissues (**A**) and hepatocytes (**B**) were harvested to detect the mRNA expression of CTGF, TGF-β and Sox9 by real-time PCR after Lv treatment for two weeks. (**C**) Protein expression of pro-fibrotic growth factors (CTGF, TGF-β) and β-catenin in hepatocytes was detected by western blotting. (**D**) The expression of pro-fibrotic growth factors (CTGF and TGF-β) in the liver was detected by immunohistochemical staining. Sj, *S. japonicum*. Scale bar, 100 μm (left) and 25 μm (right). Data represent the mean ± SEM; n = 5 (*P < 0.05).

To investigate the effects of Wnt signalling on the expression of CTGF and TGF-β, the mice infected with *S. japonicum* for 6 weeks were transfected with either Lv-DKK1-EGFP or the control vector Lv-EGFP *in vivo* by tail vein injection. Two weeks later, the liver tissues and hepatocytes were isolated to examine the expression of CTGF and TGF-β. The results showed that DKK1 reduced the mRNA levels of CTGF, TGF-β and the Wnt target gene Sox9 in liver tissues and hepatocytes (Fig. 5A,B), and the protein levels of CTGF, TGF-β (Fig. 5C,D and Supplementary Fig. 13) and β-catenin (Fig. 5C) were also decreased in the hepatocytes.

### Hepatocytes from *S. japonicum*-infected mice accelerate activation and collagen expression in HSCs relying on Wnt signalling *in vitro*

**Figure 6:**
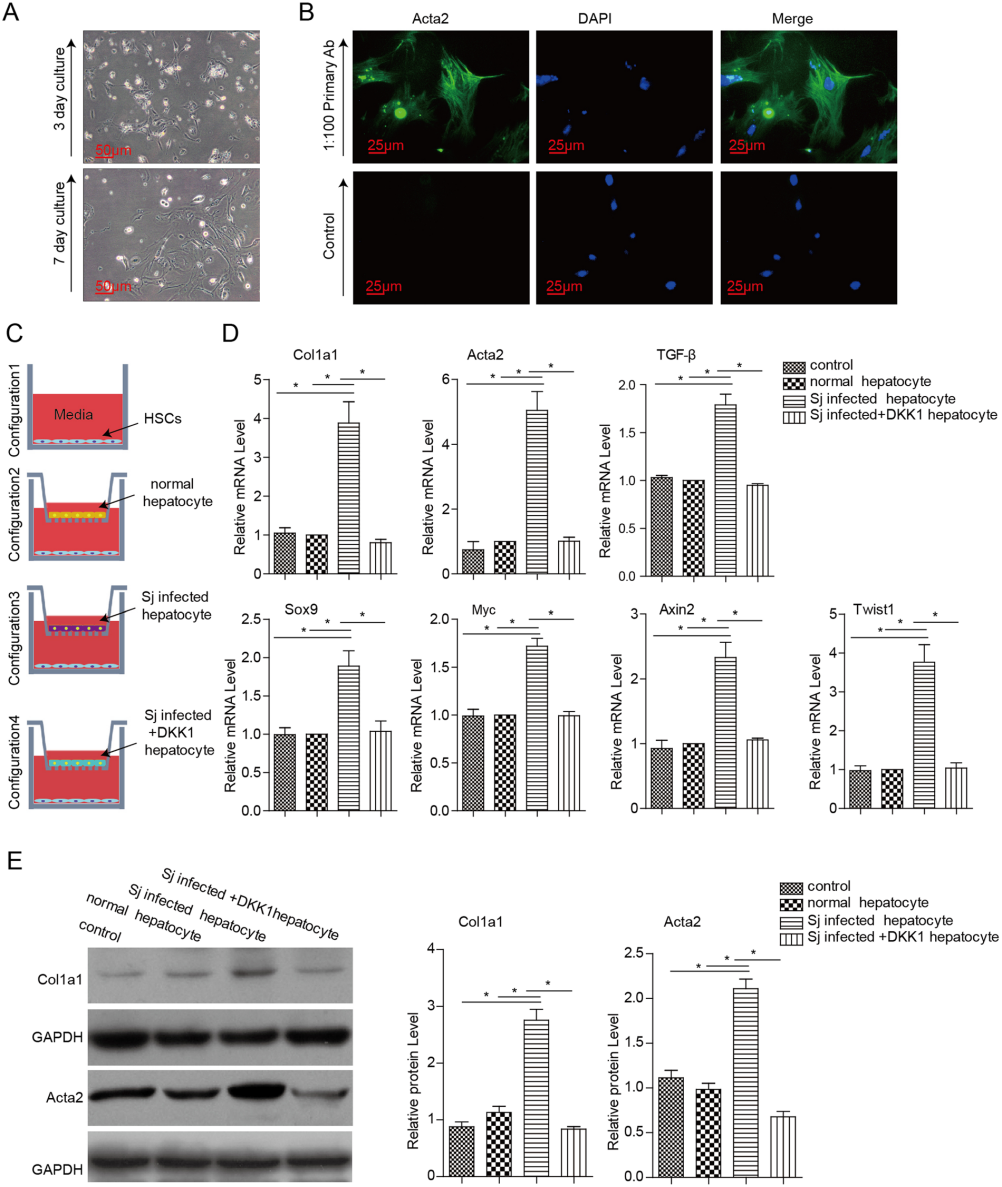
Primary hepatocytes isolated from *S. japonicum*-infected mice accelerate the activation of HSCs relying on Wnt signalling. The primary hepatocytes that were freshly isolated from normal mice and *S. japonicum*-infected mice with or without Lv-DKK1-EGFP administration were co-cultured with primary HSCs that had been cultured for 36 hours. (**A**) Phase contrast microscopic pictures of primary HSCs after 3 and 7 days of culture. (**B**) Immunofluorescence staining for α-SMA in isolated HSCs after 10 days of culture. (**C**) Pictures showing the pattern of the four-variable co-culture systems. (**D**) mRNA expression of Col1a1, α-SMA/Acta2 and TGF-β, as well as Wnt targets (Axin2, Myc, Sox9 and Twist1), was analysed after co-culture for 36 hours with hepatocytes by real-time PCR. (**E**) The protein expression of Col1a1 and α-SMA/Acta2 was analysed after co-culture for 36 hours with hepatocytes by western blotting. Scale bar, 50 μm (**A**) and 25 μm (**B**). Data represent the mean ± SEM; n = 3 (*P < 0.05).

Primary HSCs were isolated and cultured *in vitro* as demonstrated. The cellular morphology from 3 to 7 days of culture was observed (Fig. 6A), and the expression of α-SMA/Acta2 after 10 days of culture was detected (Fig. 6B). To further confirm the effects of hepatocyte-derived Wnt signalling on the activation of HSCs, primary hepatocytes freshly isolated from normal mice and *S. japonicum*-infected mice with or without Lv-Dkk1-EGFP administration were co-cultured with primary HSCs that had been cultured for 36 hours in a transwell system. Compared with HSCs grown in monoculture or co-cultured with normal hepatocytes, the mRNA expression of Col1a1, α-SMA/Acta2, TGF-β and Wnt targets (Axin2, Myc, Sox9 and Twist1) was upregulated under the conditions of co-culture with hepatocytes isolated from *S. japonicum*-infected mice, and an increase in the protein expression of Col1a1 and α-SMA/Acta2 was also detected. However, co-culture with hepatocytes from the Lv-Dkk1-EGFP-treated, *S. japonicum*-infected mice did not exhibit the same effects on the primary HSCs (Fig. 6C,D). These results demonstrate that an increase in the levels of Wnt ligands released from hepatocytes induced by *S. japonicum* infection promotes activation and collagen production in HSCs, which is an important inducible factor in schistosomal-induced liver fibrosis.

## Discussion

Liver injury caused by schistosome infection results in the formation of granulomas after the schistosome eggs become trapped in the liver. Massive deposition of the ECM is mostly found in the periportal area and leads to the characteristic pipe-stem liver fibrosis as an aberrant healing process that overrides liver regeneration^2^. The activation of HSCs and excessive production of the ECM are the central features of the pathology^2, 7^. The mechanism by which the *S. japonicum* eggs deposited in the intrahepatic microenvironment modulate liver fibrosis has yet to be defined. This study focused on exploring which signalling pathway(s) are critical to the activation of HSCs and how they affect the development of liver fibrosis.

It has been previously demonstrated that *S. mansoni* eggs are able to revert the myofibroblastic phenotype of activated HSCs back into quiescent lipid-storing cells^21^. Some studies have also shown that *S. japonicum* eggs can reduce the gene expression of α-SMA/Acta2 and Col1a1 and promote a pro-inflammatory and anti-fibrogenic phenotype rather than stimulate the activation of HSCs^22, 23^. Based on these findings, it has been implied that the activation of HSCs may rely on the increase in intrahepatic pro-fibrogenic signalling that is induced when the schistosome eggs are deposited in the liver. Thus, the change in the intrahepatic microenvironment could also act as a decisive factor affecting the activation of HSCs and the progression of schistosomiasis-induced liver fibrosis, and all of the cells of the liver may participate in this change. As the main cells involved in liver structure and function, the liver parenchymal cells may play an indispensable role in this process. Specifically, it has been suggested that damage-associated molecular patterns (DAMPs) released from dying hepatocytes may directly or indirectly promote fibrogenesis^24, 25^, and more recent studies have also implicated the inflammatory cytokines secreted from stressed hepatocytes in the promotion of fibrogenesis^24, 26^.

A recent study has confirmed that a number of Wnt and five Fz genes are expressed in hepatocytes, as well as stellate and Kupffer cells. These data indicate that the Wnt and frizzled genes expressed in the liver might play important roles in liver pathobiology via canonical or non-canonical pathways^20^. The abnormal expression of Wnt and Fz genes may contribute to liver pathology. The expression of Wnt signalling-related genes (Wnt5a, Wnt4, Fz1, Fz2, Fz7, Sox9 and Twist) has been shown to be increased in activated HSCs^27^, and Wnt signalling can enhance the activation and survival of human hepatic stellate cells^18^. In another study, the upregulation of Wnt genes (Wnt3a, Wnt10b, Wnt4, and Wnt5a) and Fz genes (Fz1, Fz2) was observed in HSCs isolated from rats with cholestatic liver fibrosis, which can be inhibited by the Wnt antagonist DKK1^17^.

In this study, we found that the expression of Wnt genes (Wnt1, Wnt2, Wnt3, Wnt3a and Wnt5a) was increased in hepatocytes isolated from mice infected with *S. japonicum*, and the protein expression of Wnt3/3a was consistent at the level of transcription. The increase in Wnt-secreted proteins may activate Wnt signalling in resident liver cells. Wnt3a is a major molecule that activates the canonical Wnt pathway^18^, and DKK1 can bind to LRP6 and inhibit Wnt-induced Fz-LRP5/6 complex formation, which is followed by suppression of the canonical Wnt pathway^17^. Therefore, we established a lentiviral vector loaded with either Wnt3a mRNA or Dkk1 mRNA and used them as transfected viruses to investigate the effects of the canonical Wnt pathway on schistosomiasis-induced liver fibrosis. The results showed that the over expression of Wnt3a exacerbates liver fibrosis, and intervention with DKK1 ameliorates the liver fibrosis. These results demonstrate that Wnt signalling plays an important role in formation of hepatic fibrosis induced by schistosome infection. Furthermore, we isolated hepatocytes from normal mice and *S. japonicum*-infected mice with or without Lv-DKK1-EGFP administration, and the primary HSCs were co-cultured with the isolated hepatocytes in a trans-well system. We observed an increase in the expression levels of Col1a1, α-SMA/Acta2, TGF-β and Wnt targets (Axin2, Myc, Sox9 and Twist1) in the HSCs that were co-cultured with hepatocytes from the *S. japonicum*-infected mice compared with the HSCs that were co-cultured with normal hepatocytes, and an increase in the protein expression of collagen α1 and α-SMA/Acta2 was also detected. However, co-culture with the hepatocytes from the Lv-DKK1-EGFP-treated, *S. japonicum*-infected mice did not have similar effects on the primary HSCs. Based on these results, we can conclude that an increase in the amount of Wnt ligands released from hepatocytes induced by *S. japonicum* infection promotes activation and collagen production in HSCs as a potential source of the critical signals that facilitate schistosomiasis-induced liver fibrosis.

Different types of damage to the liver may drive repair patterns in a variety of ways, including aberrant healing (fibrosis) and liver regeneration^28^. Liver regeneration induced by inflammatory injury during *S. japonicum* infection is poorly understood so far. It was recently reported that lncRNA-LALR1 promotes cell cycle progression and accelerates hepatocytic proliferation during liver regeneration by activating Wnt/β-catenin signalling^29^. Another independent study also demonstrated that hepatic macrophages highly express Wnt3a as a result of the phagocytosis of biological debris, and Wnt3a plays a positive role in the hepatic progenitor cell **(**HPC)-mediated regeneration of hepatocytes by activating Wnt/β-catenin signalling^30^. Therefore, it can be concluded that Wnt signalling is also crucial for liver regeneration. Our study found that the expression of Wnt genes (Wnt1, Wnt2, Wnt3, Wnt3a and Wnt5a) was upregulated, and Wnt pathway signalling in hepatocytes was active during the progression of schistosomiasis-induced liver fibrosis. However, it is also possible that this elevated Wnt signalling participated in the liver repair of the inflammatory injury caused by *S. japonicum* eggs. Our results indicate that *S. japonicum* infection upregulates the expression of hepatocellular Sox9, a target gene of Wnt signalling. Previous research has shown that hepatocytes are differentiated from Sox9-expressing precursors after acute liver damage by CCl4 treatment and bile duct ligation to promote liver regeneration^31^. An increase in the expression of Sox9 in hepatocytes may indicate that the hepatocytes can transdifferentiate into hepatic progenitor cells under the challenge of *S. japonicum* infection, and this effect may also contribute to repair of the inflammatory damage caused by egg deposition. Meanwhile, it is possible that the number of cells expressing markers of HPCs may be increased after schistosome infection and participate in liver regeneration. However, the level of liver regeneration by this functional means may be faint and covered up by the fibrosis characterized by egg granuloma formation.

TGF-β is a pleiotropic cytokine involved in cell survival, proliferation, differentiation, angiogenesis, and wound-healing response. In liver fibrosis, TGF-β is a central regulator whose functions are characterized by enhancing HSC activation and ECM synthesis, inhibiting ECM degradation and inducing epithelial-mesenchymal transition^32–36^. In the normal adult liver, sinusoidal endothelial cells and Kupffer cells have relatively high constitutive mRNA levels of TGF-β, whereas quiescent HSCs faintly express TGF-β, and hepatocytes essentially express none^37, 38^. After liver injury, certain inflammatory and mesenchymal cells, including HSCs, are the principal source of TGF-β production. However, growing evidence has also provided support for the ability of parenchymal hepatocytes to produce TGF-β, both in chronic liver disease and cultured hepatocytes^39, 40^. It is believed that the release of TGF-β by necrotic hepatocytes may be one of the first signals to activate the adjacent quiescent HSCs, resulting in their transdifferentiation into proliferative, fibrogenic, and contractile myofibroblasts^32^. In our study, the expression of TGF-β was increased in hepatocytes after *S. japonicum* infection, and TGF-β was more frequently detected in the hepatocytes that were close to egg granulomas. These results suggest that hepatocytes play an important role in schistosomiasis-induced liver fibrosis as a source of fibrotic signals. The levels of hepatocyte-derived CTGF are usually low, and elevated levels of hepatic CTGF have been observed in patients with liver fibrosis and in experimental animal models of liver fibrosis. In the fibrotic liver, CTGF is produced by multiple types of cells, including hepatocytes, fibroblasts, myofibroblasts, HSCs, endothelial cells, mononuclear cells, inflammatory cells, and bile duct epithelial cells^41–45^. CTGF can promote proliferation, survival, migration, adhesion, and ECM production in activated hepatic stellate cells and consequently boosts hepatic fibrosis^41, 45–47^. In this study, the level of CTGF was found to be upregulated in hepatocytes after *S. japonicum* infection, and CTGF was often detected in hepatocytes that were adjacent to areas containing egg granulomas. Together, these results suggest that CTGF produced by hepatocytes may play an important role in schistosomiasis-induced liver fibrosis. One study has recently identified hepatocytes as a newly recognized and important source of TGF-β-regulated CTGF secretion^48^. Our results were not sufficient to support this idea in schistosomiasis-induced liver fibrosis, although hepatocytes are most likely involved in promoting hepatic fibrosis in schistosomiasis-induced liver fibrosis. The elevated TGF-β and CTGF levels provide obvious evidence of this notion.

In our study, Lv-DKK1 was injected into mice infected with *S. japonicum*. The suppression of the Wnt pathway was accompanied by much lower levels of TGF-β and CTGF in hepatocytes, which indicates that activation of the Wnt pathway triggers the production of TGF-β and CTGF in hepatocytes during schistosomiasis-induced liver fibrosis. Combined with previous reports, these results imply that Wnt-secreted proteins from hepatocytes not only directly activate HSC but also stimulate the expression of TGF-β and CTGF by activating the Wnt pathway itself to enhance liver fibrosis. It has been reported that TGF-β and Wnt ligands can regulate each other’s expression during early development. A study of chicken embryonic development revealed that Wnt8c can induce the expression of Nodal, a TGF-β family member, in a β-catenin-dependent manner^49^. Another study involving Xenopus showed that Wnt8 expression was regulated by TGF-β family members^50^. However, the mechanism of how activation of the Wnt pathway affects the expression of TGF-β and CTGF in hepatocytes needs to be further studied.

**Figure 7:**
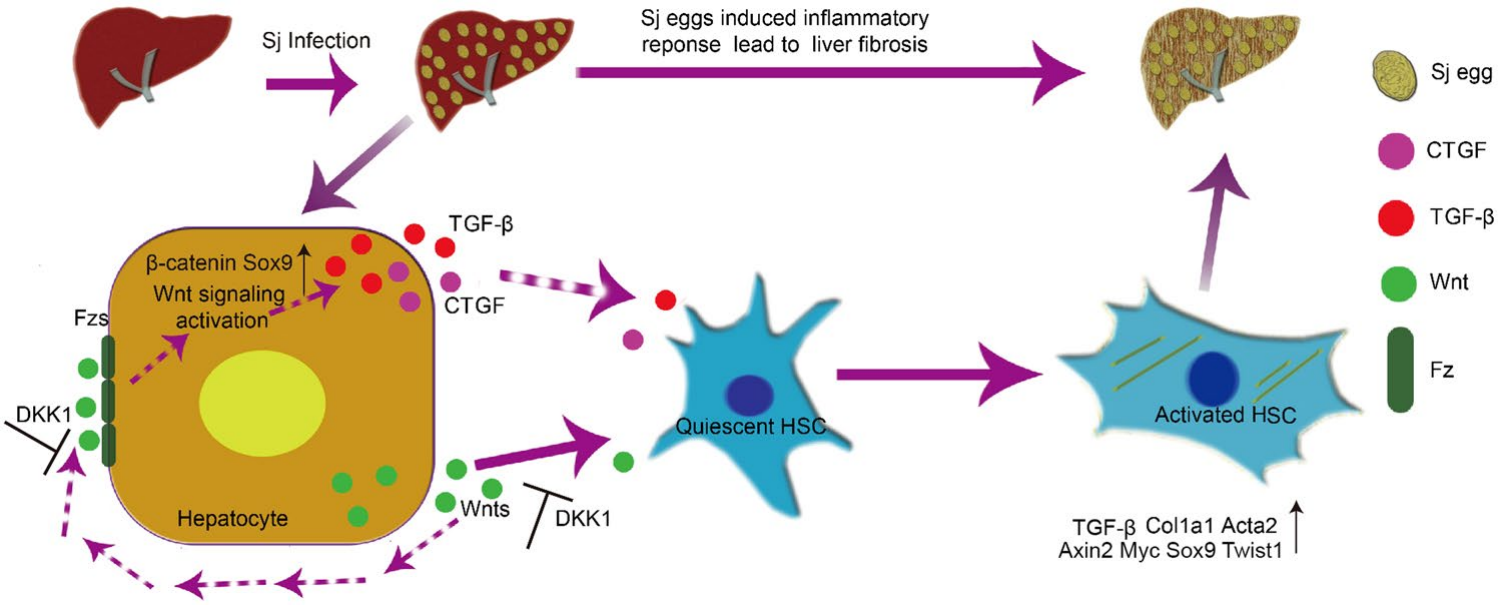
Enhanced Wnt signalling in hepatocytes contributes to liver fibrosis induced by *S. japonicum* infection. During the development of Schistosomiasis liver fibrosis, Enhanced Wnt signalling of hepatocytes promotes liver fibrogenesis. In addition, *S. japonicum* infection accelerates the expression of pro-fibrogenic factors TGF-β and CTGF in hepatocytes, which is controlled by Wnt signalling.

It is widely accepted that the pathology of hepatic schistosomiasis arises when schistosome eggs become lodged in the liver, which evokes an IL-4- and IL-13-mediated dominant CD4^+^ Th2 immune response^1^. This response leads to the development of granulomas and fibrosis, and eosinophils, neutrophils, macrophages, hepatic stellate cells, and lymphocytes have been identified as major cellular contributors to these events^4^. However, little attention has been paid to the effects of hepatocytes on the progression of liver fibrosis. In our study (Fig. 7), we uncovered a previously unknown pro-fibrogenic function of hepatocytes in which Wnt signalling derived from hepatocytes appears to promote liver fibrogenesis triggered by schistosomiasis. In addition, we have also shown that *S. japonicum* infection accelerates the hepatocellular expression of the pro-fibrogenic factors TGF-β and CTGF, which may be regulated by Wnt signalling. However, the effects of Wnt signalling may be diverse in different pathologic states. These differences depend much more on changes to the intrahepatic microenvironment in different liver diseases than on Wnt signalling itself.

## Materials and Methods

### Ethics Statement

The animal experiments were performed in accordance with the guidelines of the Chinese Council on Animal Care, and the study was approved by the Tongji Medical College Committee (Wuhan, China).

### Mice and Parasitic Infections

Eight-week-old female BALB/c mice were purchased from the Hubei Provincial Center for Disease Control and Prevention (Wuhan, China) and maintained in a specific pathogen-free microenvironment. *Oncomelania hupensis* snails were provided by the Nanjing Institute of Schistosomiasis Prevention and Control (Nanjing, China). Anesthetized mice were percutaneously infected with 25 cercariae of *S. japonicum*.

### Construction of Lentivirus and Gene Transfection *in vivo*

The cDNA of mouse Wnt3a or DKK1 were subcloned into the lentiviral vector pLV (Exp)-Neo-EF1A > EGFP to generate pLV[Exp]-Neo-EF1A > mDkk1[NM_010051.3]:IRES:EGFP or pLV[Exp]-Neo-EF1A > ORF_1059 bp(mWnt3a):IRES:EGFP (Cyagen Biosciences Inc., Guangzhou, China). The recombinant lentiviruses and control lentiviruses were intravenously injected at a dose of 1 × 10^8^ PFU per mouse in 150 μl of physiological saline. Two weeks later, the liver tissues were processed as frozen sections, and the expression of EGFP was observed using a fluorescence microscope.

### Isolation of Mouse Primary Hepatocytes and HSCs

The main solutions were prepared according to the following formulas:

Solution I: 8 g/L NaCl, 0.4 g/L KCl, 0.06 g/L KH_2_PO_4_, 0.13 g/L Na_2_HPO_4_•12H_2_O, 0.07 g/L EGTA, 0.35 g/L NaHCO_3_, pH = 7.4.

Solution II: 8 g/L NaCl, 0.4 g/L KCl, 0.13 g/L Na_2_HPO_4_•12H_2_O, 10 mmol/L HEPES, 0.14 g/L CaCl_2_, 0.35 g/L NaHCO_3_, pH = 7.4.

Solution III: 8 g/L NaCl, 0.4 g/L KCl, 0.13 g/L Na_2_HPO_4_•12H_2_O, 0.14 g/L CaCl_2_, 0.35 g/L NaHCO_3_, 0.2 g/L collagenase IV (Gibco, USA), pH = 7.4.

The primary hepatocytes were isolated using a 2-step collagenase perfusion (HEPES, pH 7.4) and low-speed centrifugations. Briefly, the liver was successively perfused with Solution I, Solution II and Solution III through the hepatic portal vein, and the liver tissues were then triturated and suspended in PBS. After filtration through a 200-gauge mesh, the parenchymal cells were separated from the collected cell suspension by three centrifugations at 100 × *g*, 65 × *g* and 40 × *g* for 3 min each. After removal of the hepatocytes, the remaining supernatant was centrifuged at 500 × *g* for 5 min to pellet the non-parenchymal cells. The pellet was then resuspended in DMEM and added to a 20%*/*40% Percoll density gradient, followed by centrifugation at 900 × *g* for 15 min. The HSCs that accumulated at the interface of the 20% and 40% Percoll density gradient were collected and resuspended in DMEM, followed by centrifugation at 500 × *g* for 5 min to remove the remaining Percoll solution. The collected cells that were resuspended in DMEM containing 20% foetal bovine serum (FBS, Gibco) were then plated, and the medium was changed every 48 hours. When the cells were 70% confluent in the 6-well plate, the HSCs were co-cultured with freshly isolated hepatocytes.

### Histology and Immunohistochemical Staining

The right lobes of the liver were harvested and fixed in 4% paraformaldehyde. These liver tissues were embedded in paraffin and sliced into 4-μm sections, followed by dewaxing and rehydration. The histological sections were then either stained with haematoxylin and eosin (HE) for pathologic observation or subjected to Masson’s trichrome staining for evaluation of collagen deposition. Five fields were randomly selected from each sample for analysis, and the ratio of the area occupied by collagen fibres to the total area was quantified using the Mshot Image Analysis System (Guangzhou Micro-shot Technology Co., Ltd, China). Some sections were incubated with primary antibodies against Wnt3/3a (1:200, Abcam, Cambridge, UK), β-catenin (1:200, Abcam, Cambridge, UK), Sox9 (1:200, Abcam, Cambridge, UK), Col1a1 (1:200, Abcam, Cambridge, UK), desmin (1:200, Abcam, Cambridge, UK), α-SMA/Acta2 (1:200, Abcam, Cambridge, UK), CTGF (1:200, Abcam, Cambridge, UK) or TGF-β (1:200, Abcam, Cambridge, UK) at 4 °C for 12 hours, followed by washing and incubation with HRP-conjugated goat anti-rabbit secondary antibodies (1:500; Earthox, USA) for 1 hour at 25 °C. The sections were visualized with 3,3′-diaminobenzidine and counterstained with haematoxylin for microscopic examination. For fluorescent staining, the hepatocytes or HSCs were incubated with primary antibodies against either albumin (1:200, Abcam, Cambridge, UK) or α-SMA/Acta2 (1:200, Abcam, Cambridge, UK) at 4 °C for 12 hours after fixation in 4% paraformaldehyde and permeabilization with 0.15% Triton X-100. The cells were then incubated with fluorescein isothiocyanate (FITC)-conjugated anti-mouse IgG (1:200; Earthox, USA) at 25 °C for 4 hours. The nuclei were stained with DAPI. Fluorescent images were visualized and captured using an inverted fluorescence microscope (MF53 Mercury Guangzhou, China).

### Western blotting

Liver tissues or freshly isolated hepatocytes were lysed in RIPA Lysis buffer (Beyotime, China). Next, 40 μg of proteins were suspended in sample buffer and boiled at 95 °C for 5 min, followed by fractionation using 10% sodium dodecyl sulphate polyacrylamide gel electrophoresis. The proteins were transferred to PVDF membranes and then blocked with 5% non-fat milk in Tris-buffered saline with Tween 20 (TBST) before immune detection with the following antibodies: Wnt3/3a (1:1000, Abcam, Cambridge, UK), β-catenin (1:1000, Abcam, Cambridge, UK), Sox9 (1:1000, Abcam, Cambridge, UK), CTGF (1:1000, Abcam, Cambridge, UK), TGF-β (1:1000, Abcam, Cambridge, UK), Col1a1 (1:1000, Abcam, Cambridge, UK), desmin (1:1000, Abcam, Cambridge, UK), α-SMA/Acta2 (1:1000, Abcam, Cambridge, UK) and GAPDH (1:1000; CST, Boston, USA). After incubation with the primary antibodies overnight at 4 °C, the membranes were incubated with HRP-conjugated secondary goat anti-rabbit antibodies (1:3000; Earthox, San Francisco, USA) for 1 hour at 25 °C. The proteins were then detected with enhanced chemiluminescent substrate (ECL; Millipore, Billerica, MA, USA), and the band densities were analysed using Image Lab 4.1 and standardized according to the GAPDH band.

### Real-time PCR

Total RNA was extracted from the mouse livers and hepatocytes using Trizol reagent (Invitrogen, USA). The cDNAs were synthesized using a reverse transcription kit (ReverTra Ace qPCR RT Kit, TOYOB, Japan), and real-time PCR was performed using SYBR green master mix (SYBR GREEN PCR, TOYOB, Japan) on a MyiQTM2 (BIORAD, USA). GAPDH was used to normalize the real-time PCR data. The following primer sequences were used: Wnt1-s, 5′CGAGGCTGCCGAGAAACA 3′ and 5′ GCCAAAGAGGCGACCAAAA 3′; Wnt2, 5′ GAAGTAGTCGGGAATCGG 3′ and 5′ CATCCTTGCCTTTCCTCT3′; wnt3a, 5′ ACCACCGTCAGCAACAGC 3′ and 5′ GCGTGTCACTGCGAAAGC 3′; wnt5a, 5′ CAGGTCAACAGCCGCTTCAAC 3′ and 5′ACAATCTCCGTGCACTTCTTGC 3′; DKK1, 5′AGCCAGTGCCACCTTGA3′ and 5′ TTGTTCCCGCCCTCATA 3′; Fz1, 5′ ATGACGGCACCAAGACAGA 3′ and 5′ GGCAAGGGATGGCATAACTC 3′; CTGF, 5′ TGTGAAGACATACAGGGCTAAG 3′ and 5′ ACAGTTGTAATGGCAGGCAC 3′; Sox9, 5′ GCGAACGCACATCAAGACG 3′ and 5′ GTAAGTGAAGGTGGAGTAGAGCC 3′; GAPDH, 5′ GTGTTTCCTCGTCCCGTAG 3′ and 5′ATGGCAACAATCTCCACTTT 3′; Wnt3, 5′TGCCAGCATCAGTTCCG3′ and 5′TGACTGCGAAGGCGACA3′; TGF-β, 5′ CCCACTGATACGCCTGAG 3′ and 5′ GGGCTGATCCCGTTGAT 3′; Fz5, 5′ACACGCAGGACGAAGCAG3′ and 5′TGTGGTAGTCAGGCAAACAGAT3′; Axin2, 5′CAACGACAGCGAGTTATCC3′ and 5′GTTCCACAGGCGTCATCT3′; Myc, 5′GACTGTATGTGGAGCGGTTTC3′ and 5′CGTTGAGCGGGTAGGGA3′; Twist1, 5′CAGCGGGTCATGGCTAACG3′ and 5′CAGGACCTGGTACAGGAAGTCGA3′.

### Statistical Analysis

Data are presented as the means ± SEM of several experiments. Statistical significance was assessed by Student’s *t*-test or one-way ANOVA followed by Tukey’s *t*-test. A *P* value of less than 0.05 was considered statistically significant.

## Electronic supplementary material

Supplementary information
