# Supplementary material for: Enhanced Wnt Signalling in Hepatocytes is Associated with Schistosoma japonicum Infection and Contributes to Liver Fibrosis
Source: Sci Rep. 2017 Mar 22;7:230. doi: 10.1038/s41598-017-00377-4 (PMC5428310; doi:10.1038/s41598-017-00377-4)
Supplement: Supplementary file 1 — Supplementary information [file 41598_2017_377_MOESM1_ESM.pdf]

Enhanced Wnt Signalling in Hepatocytes is Associated with *Schistosoma japonicum* Infection and Contributes to Liver Fibrosis

Qi Wang, Xin Chou, Fei Guan, Zhengming Fang, Shengjun Lu, Jiahui Lei, Yonglong Li, Wenqi Liu*

Department of Parasitology, School of Basic Medicine, Tongji Medical College, Huazhong University of Science and Technology, Wuhan, Hubei, P.R. China

Correspondence and requests for materials should be addressed to W.L. (Email: liu_wq2002cn@hotmail.com)

Supplementary Figure

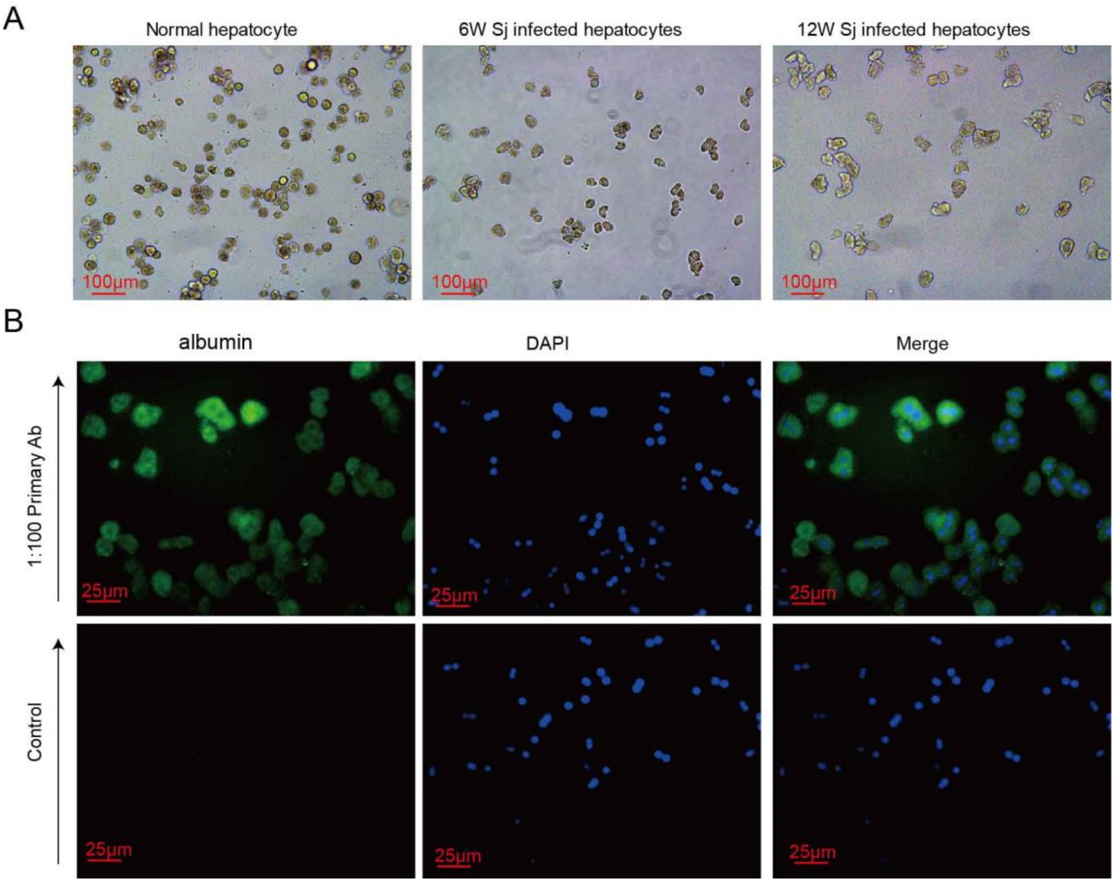

Supplementary Figure 1. Successful isolation of hepatocytes.

Primary hepatocytes were isolated from normal mice and mice infected with *S. japonicum* for 6 or12 weeks. (A) Phase contrast microscopic pictures of freshly isolated hepatocytes. (B) The purity of the freshly isolated hepatocytes was detected by immunofluorescence analysis using a FITC-conjugated anti-albumin antibody. Scale bar, 100 μm (A) and 25 μm (B)

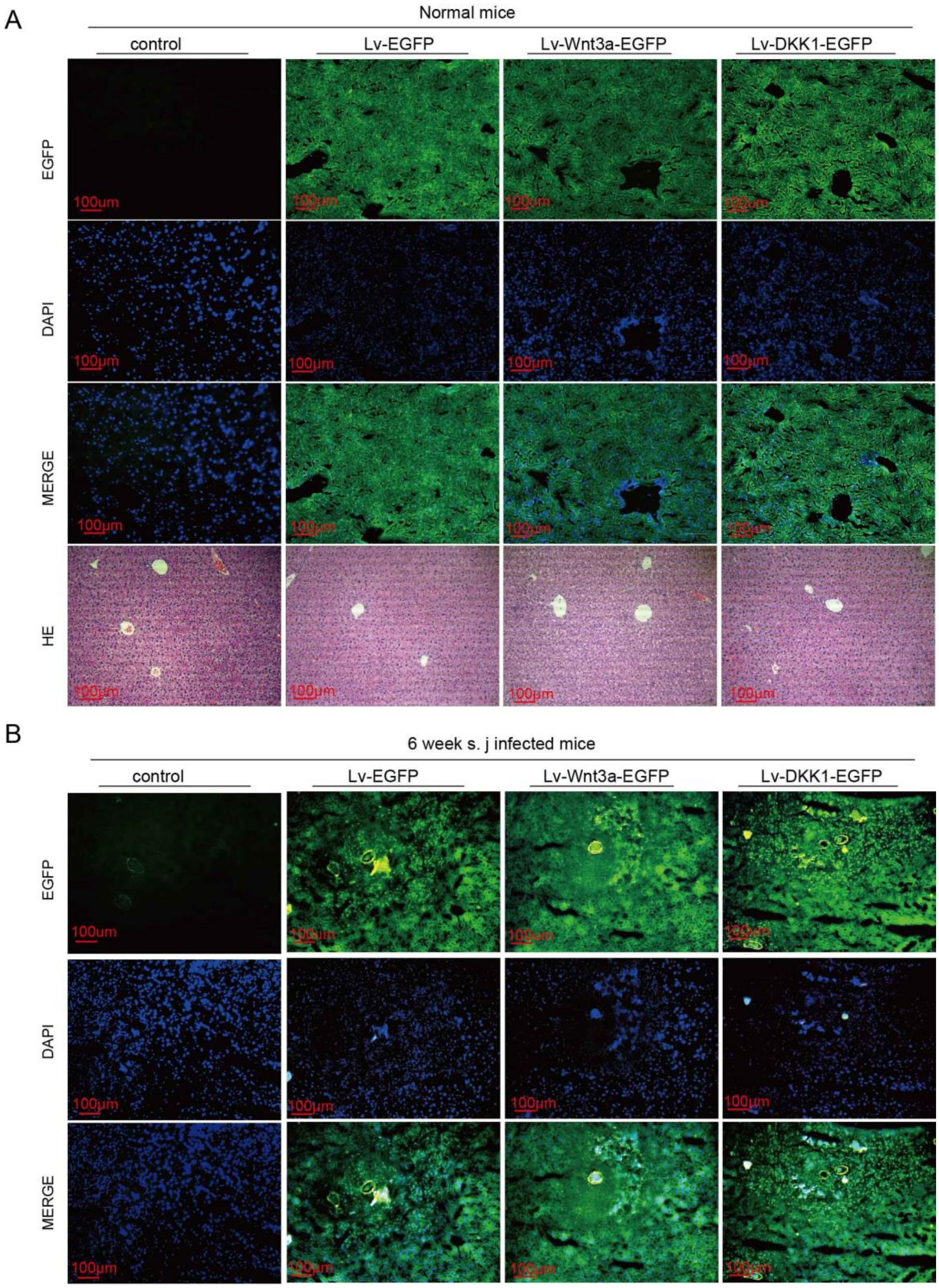

Supplementary Figure 2. Successful transfection and expression of recombinant lentiviruses (Lv-EGFP, Lv-Wnt3a-EGFP and Lv-Dkk1-EGFP) in the livers of mice infected with *S. japonicum*.

Either Lv-EGFP, Lv-Wnt3a-EGFP or Lv-Dkk1-EGFP was injected via the tail vein into

normal mice or mice that had been infected with *S. japonicum* for 6 weeks. Two weeks later, clear green fluorescence was observed in the frozen sections of the liver tissues (A B), and HE staining was used to observe the pathological changes in the liver (A). Sj, *S. japonicum*. Scale bar, 100 μm.

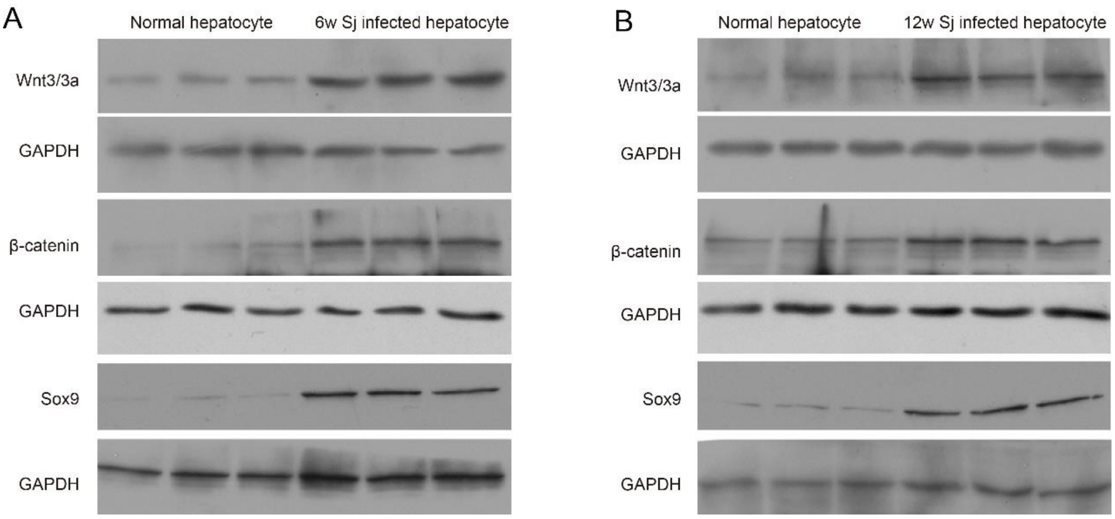

Supplementary Figure 3 (response to Fig1. C). Primary hepatocytes were isolated from normal mice and mice with *S. japonicum* infection. (A B) The expression of Wnt3/3a, β-catenin, and Sox9 was detected by western blotting 6 or 12 weeks p.i..

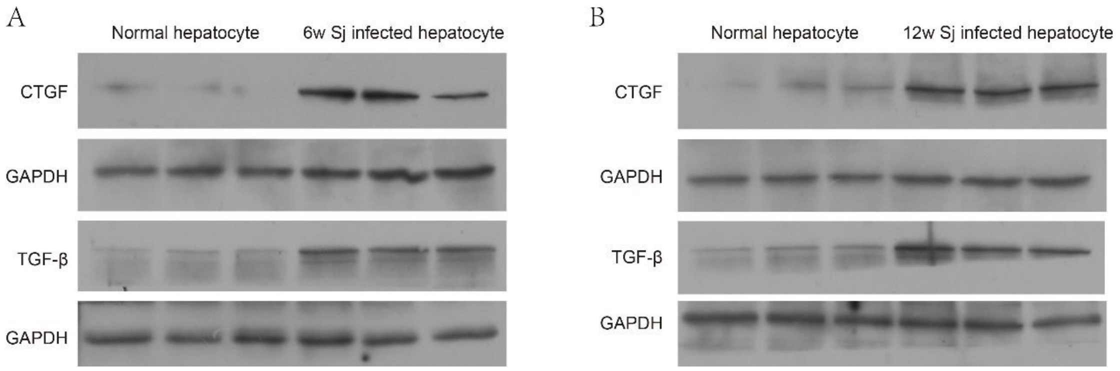

Supplementary Figure 4 (response to Fig2. B). Primary hepatocytes were isolated from normal mice and mice with *S. japonicum* infection. (A B) The expression of CTGF and TGF-β was detected by western blotting 6 or 12 weeks p.i..

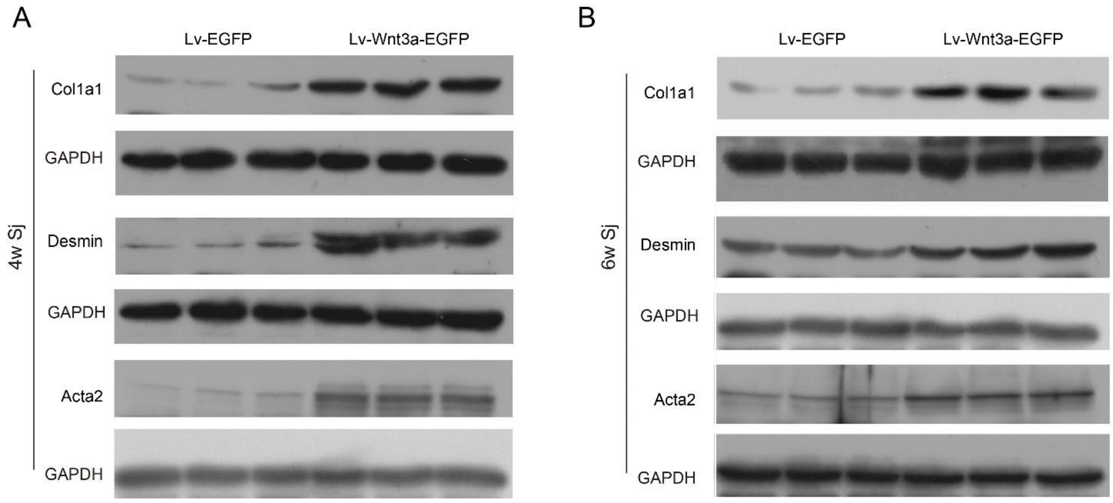

Supplementary Figure 5 (response to Fig3. C). Mice received either Lv-EGFP or Lv-Wnt3a-EGFP 4 or 6 weeks after *S. japonicum* infection. The hepatic expression of fibrotic markers (Col1a1, Desmin, and α-SMA/Acta2) was assessed by western blotting.

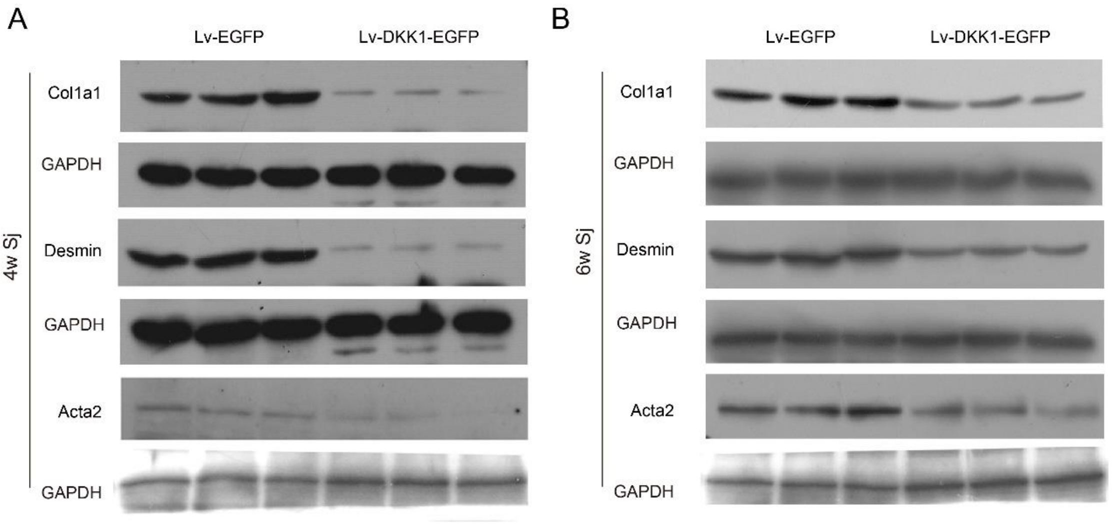

Supplementary Figure 6 (response to Fig4. C). Lv-DKK1-EGFP was injected via the tail vein to suppress Wnt signalling in the liver 4 or 6 weeks after *S. japonicum* infection. (A B) Fibrotic markers (Col1a1, Desmin, and α-SMA/Acta2) in the liver were assessed by western blotting.

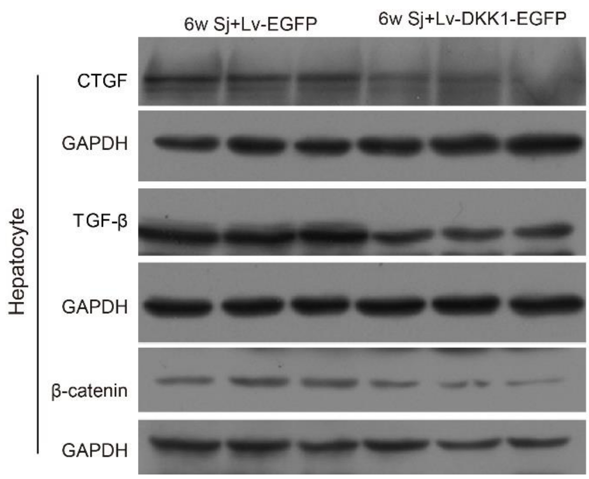

Supplementary Figure 7 (response to Fig5. C). Lv-DKK1-EGFP was injected via the tail vein to suppress after *S. japonicum* infection for 6 weeks. The hepatocytes were harvested to detect the pro-fibrotic growth factors (CTGF, TGF-β) and β-catenin in hepatocytes by western blotting.

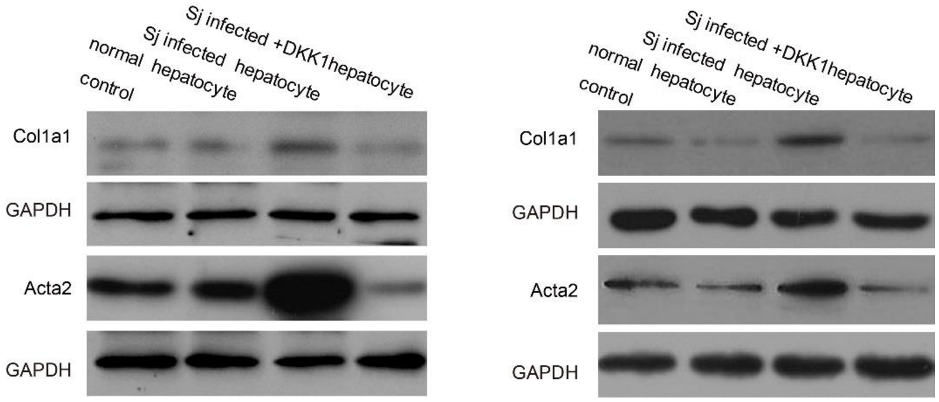

Supplementary Figure 8 (response to Fig6. E). The primary hepatocytes isolated from normal mice and *S. japonicum*-infected mice with or without Lv-DKK1-EGFP administration were co-cultured with primary HSCs. The protein expression of Col1a1 and α-SMA/Acta2 of HSCs was analysed after co-culture by western blotting.

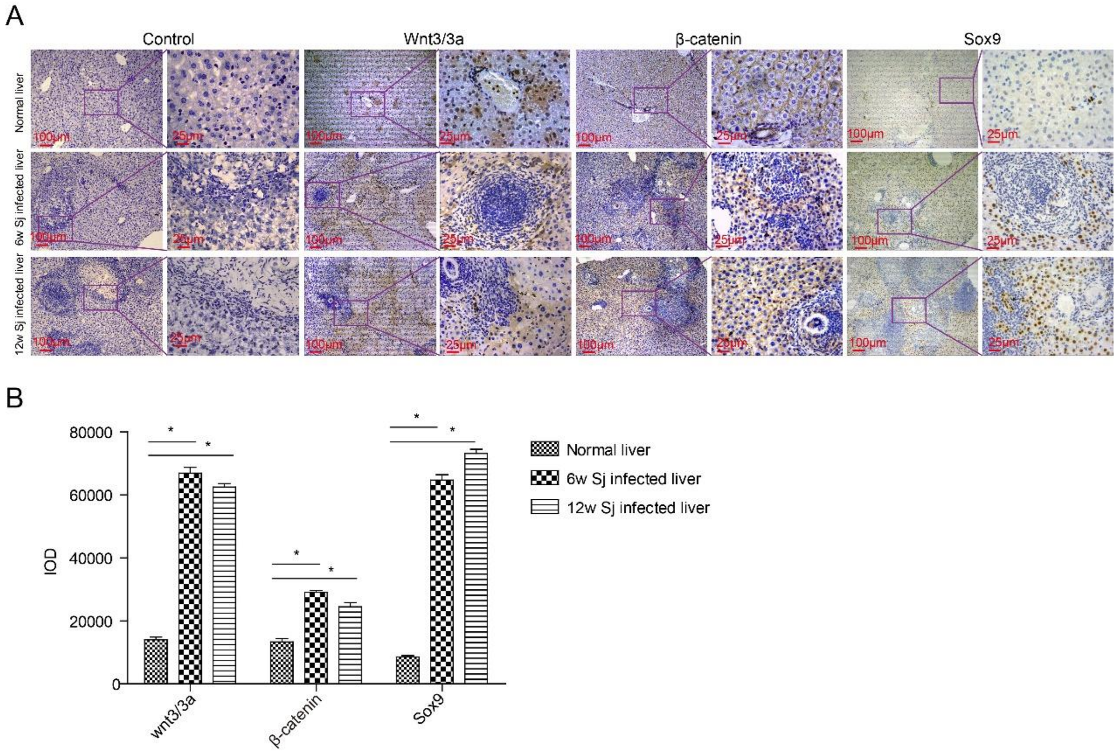

Supplementary Figure 9 (response to Fig1. C). (A) Liver sections from normal mouse and *S. japonicum* infected mouse were immunohistochemically stained for Wnt3/3a, β-catenin, and Sox9. (B)The integrated optical density (IOD) of Wnt3/3a, β-catenin, and Sox9 was assessed by Image-Pro Plus. Data represent the mean ±SEM; n= 5 (* $P < 0.05$).

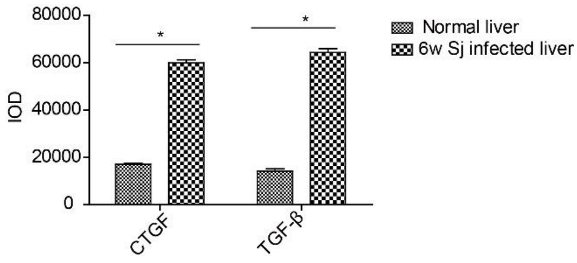

Supplementary Figure 10 (response to Fig2. C). Liver sections from normal mouse and *S. japonicum* infected mouse were immunohistochemically stained for CTGF and TGF-β. The IOD of CTGF and TGF-β was assessed by Image-Pro Plus. Data represent the mean ± SEM; n= 5 (* P < 0.05).

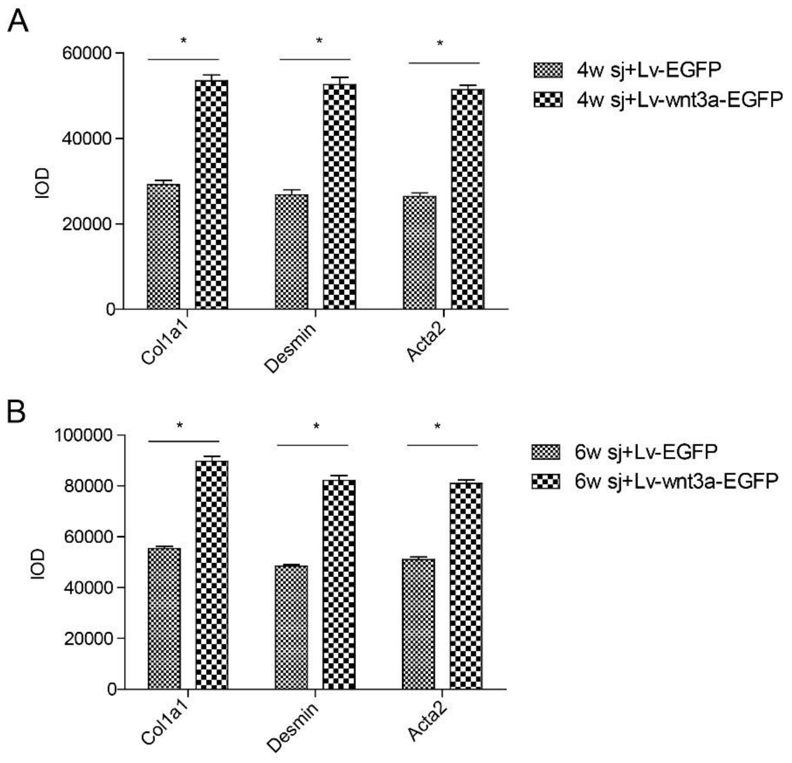

Supplementary Figure 11 (response to Fig3. B**).** Mice received either Lv-EGFP or Lv-Wnt3a-EGFP after *S. japonicum* infection for 4 (A) or 6 weeks (B). Hepatic expression of fibrotic markers (Col1a1, desmin, and α-SMA/Acta2) was assessed by immunohistochemical staining. (A B) The IOD of Col1a1, Desmin, and α-SMA/Acta2 was assessed by Image-Pro Plus. Data represent the mean ± SEM; n= 5 (* $P < 0.05$).

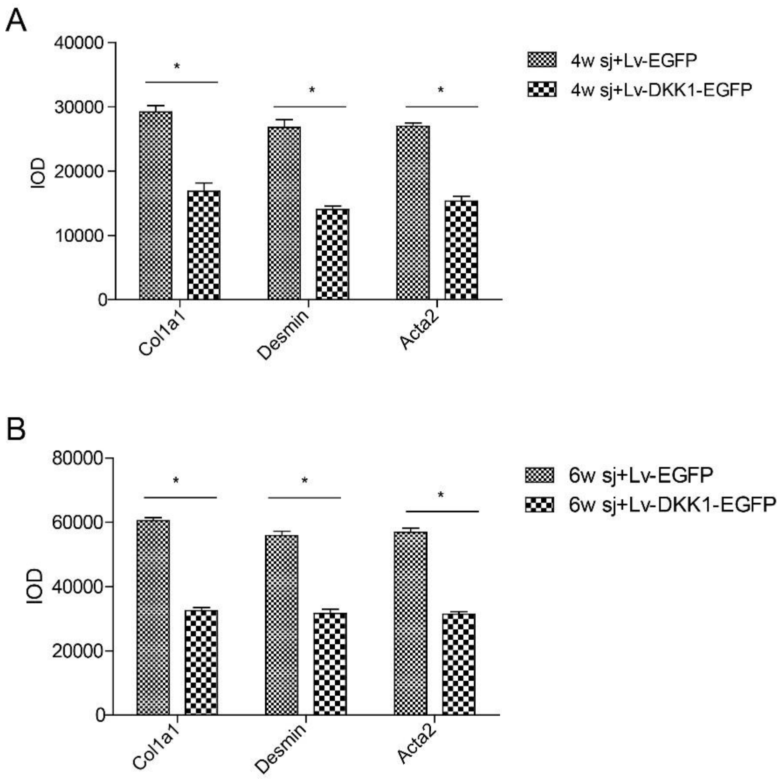

Supplementary Figure 12 (response to Fig4. B**).** Mice received either Lv-EGFP or Lv-DKK1-EGFP after *S. japonicum* infection for 4 (A) or 6 weeks (B). Hepatic expression of fibrotic markers (Col1a1, desmin, and α-SMA/Acta2) was assessed by immunohistochemical staining. (A B) The IOD of Col1a1, Desmin, and α-SMA/Acta2 was assessed by Image-Pro Plus. Data represent the mean ± SEM; n= 5 (* $P < 0.05$).

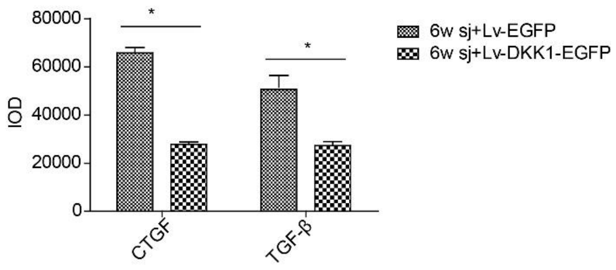

Supplementary Figure 13 (response to Fig5. D**).** Mice received either Lv-EGFP or Lv-DKK1-EGFP after *S. japonicum* infection for 6 weeks. The expression of pro-fibrotic growth factors (CTGF and TGF-β) in the liver was detected by immunohistochemical staining. The IOD of CTGF and TGF-βwas assessed by Image-Pro Plus. Data represent the mean ± SEM; n= 5 (* $P < 0.05$).

Supplementary material

The cDNA sequence of mouse Wnt3a used in present study: atggct cctctcggat acctcttagt gctctgcagc ctgaagcagg ctctgggcag ctacccgatc tggtggtcct tggctgtggg accccagtac tcctctctga gcactcagcc cattctctgt gccagcatcc caggcctggt accgaagcag ctgcgcttct gcaggaacta cgtggagatc atgcccagcg tggctgaggg tgtcaaagcg ggcatccagg agtgccagca ccagttccga ggccggcgtt ggaactgcac caccgtcagc aacagcctgg ccatctttgg ccctgttctg gacaaagcca cccgggagtc agcctttgtc catgccatcg cctccgctgg agtagctttc gcagtgacac gctcctgtgc agagggatca gctgctatct gtgggtgcag cagccgcctc cagggctccc caggcgaggg ctggaagtgg ggcggctgta gtgaggacat tgaatttgga ggaatggtct ctcgggagtt tgccgatgcc agggagaacc ggccggatgc ccgctctgcc atgaaccgtc acaacaatga ggctgggcgc caggccatcg ccagtcacat gcacctcaag tgcaaatgcc acgggctatc tggcagctgt gaagtgaaga cctgctggtg gtcgcagccg gacttccgca ccatcgggga tttcctcaag gacaagtatg acagtgcctc ggagatggtg gtagagaaac accgagagtc tcgtggctgg gtggagaccc tgaggccacg ttacacgtac ttcaaggtgc cgacagaacg cgacctggtc tactacgagg cctcacccaa cttctgcgaa cctaaccccg aaaccggctc cttcgggacg cgtgaccgca cctgcaatgt gagctcgcat ggcatagatg ggtgcgacct gttgtgctgc gggcgcgggc ataacgcgcg cactgagcga cggagggaga aatgccactg tgttttccat tggtgctgct acgtcagctg ccaggagtgc acacgtgtct atgacgtgca cacctgcaag tag.

The cDNA sequence of mouse DKK1 used in present study: atg atggttgtgt gtgcagcggc agctgtccgg ttcttggccg tgtttacaat gatggctctc tgcagcctcc ctctgctagg agccagtgcc accttgaact cagttctcat caattccaac gcgatcaaga acctgccccc accgctgggt ggtgctgggg ggcagccggg ctctgctgtc agtgtggcgc cgggagttct ctatgagggc gggaacaagt accagactct tgacaactac cagccctacc cttgcgctga agatgaggag tgcggctctg acgagtactg ctccagcccc agccgcgggg

cagccggcgt cggaggtgta cagatctgtc tggcttgccg aaagcgcagg aagcgctgca tgaggcacgc tatgtgctgc cccgggaact actgcaaaaa tggaatatgc atgccctctg accacagcca ttttcctcga ggggaaattg aggaaagcat cattgaaaac cttggtaatg accacaacgc cgccgcgggg gatggatatc ccagaagaac cacactgact tcaaaaatat atcacaccaa aggacaagaa ggctccgtct gcctccgatc atcagactgt gccgcagggc tgtgttgtgc aagacacttc tggtccaaga tctgtaaacc tgtccttaaa gaaggtcagg tgtgcaccaa gcacaaacgg aaaggctccc acgggctgga gatattccag cgctgttact gcggggaagg cctggcttgc aggatacaga aagatcacca tcaagccagc aattcttcta ggctccacac ctgccagaga cactaa.
